# Keratinases as Versatile Enzymatic Tools for Sustainable Development

**DOI:** 10.3390/biom11121900

**Published:** 2021-12-18

**Authors:** Marcin Sypka, Iga Jodłowska, Aneta M. Białkowska

**Affiliations:** Institute of Molecular and Industrial Biotechnology, Lodz University of Technology, Stefanowskiego 2/22, 90-537 Lodz, Poland; marcin.sypka@dokt.p.lodz.pl (M.S.); iga.jodlowska@dokt.p.lodz.pl (I.J.)

**Keywords:** keratinases, keratinolytic microorganisms, keratin waste, sustainable development

## Abstract

To reduce anthropological pressure on the environment, the implementation of novel technologies in present and future economies is needed for sustainable development. The food industry, with dairy and meat production in particular, has a significant environmental impact. Global poultry production is one of the fastest-growing meat producing sectors and is connected with the generation of burdensome streams of manure, offal and feather waste. In 2020, the EU alone produced around 3.2 million tonnes of poultry feather waste composed primarily of keratin, a protein biopolymer resistant to conventional proteolytic enzymes. If not managed properly, keratin waste can significantly affect ecosystems, contributing to environmental pollution, and pose a serious hazard to human and livestock health. In this article, the application of keratinolytic enzymes and microorganisms for promising novel keratin waste management methods with generation of new value-added products, such as bioactive peptides, vitamins, prion decontamination agents and biomaterials were reviewed.

## 1. Introduction

In order to reduce anthropological pressure on the environment and to mitigate threats connected with progressing climate change, many governmental, business, social and scientific organizations proposed actions and concepts that could facilitate positive changes and the implementation of novel models and technologies in present and future economies. Sustainable development (SD) is seen as one of the most ambitious and broad models formulated to date [1]. The United Nations seventeen SD goals (SDGs) with their targets and indicators (as a part of the 2030 Agenda) were designed to address and to connect environmental protection issues with economic growth and social advancement [2,3]. Since the framework’s announcement, many have identified proposed specific focus points, actions and transformations needed for successful implementation of SDGs on national and global levels [1,4,5]. Decarbonization of energy production systems, limitation of greenhouse-gas emissions, combating land, water and air pollutions as well as securing access to food and fresh water without compromising natural habitats and loss of biodiversity, are thought to be the main challenges and areas requiring heavy transformations in achieving environmental sustainability [1].

The food industry, with dairy and meat production in particular, is known to pose a significant environmental impact consisting of land and water use, carbon dioxide, nitrogen oxide and methane emissions, soil erosion, eutrophication of water reservoirs and considerable amounts of generated waste. Poultry meat production is not an exception [6,7,8].

In 2020, The European Union (EU-27) produced over 13.5 million tonnes of poultry meat, with Poland (2.6 million tonnes), Spain (1.7 million tonnes), France (1.7 million tonnes), Germany (1.6 million tonnes) and Italy (1.4 million tonnes) as the main producers. In the EU, the poultry industry is dominated by production of broiler chicken meat (82%), followed by turkey (14%), duck (3%) and others (1%) [9]. Global poultry production is one of the fastest-growing meat producing sectors and is connected with generation of burdensome streams of manure, offal and feather waste [10,11,12]. It is estimated that in 2020, the EU’s poultry meat production of 13.5 million tonnes created around 3.2 million tonnes of feather waste [13].

Poultry feather waste is mainly composed of keratin—a protein biopolymer [14]. Thanks to its complex structure, keratin is characterized by its high stability, durability and resistance to hydrolysis by common proteolytic enzymes such as pepsin, trypsin and chymotrypsin [15]. Traditionally keratin waste is disposed in the landfills, incinerated or converted into animal feed, using physical and chemical treatments [14]. If not managed properly, keratin waste, including feathers, can significantly affect ecosystems, contributing to environmental pollution, and pose a serious hazard to human and livestock health [16].

As the sustainability of the global economy, including closing of production cycles, decarbonization and bioeconomy development, has become of a great need, rational, unconventional and innovative keratin waste management methods are now subject of extensive international research, with India, China and Brazil, followed by South Korea, the United States, Egypt and Japan leading in research productivity of that field [12,17].

Keratinolytic microorganisms and keratinases can be applied in various industries, such as leather, textile, chemical, pharmaceutical, food and nutrition industries, as well as in biotechnology and environmental protection [18,19,20].

## 2. Keratin Characterization

Keratins are a heterogeneous family of proteins and the third most abundant biomass in nature, after chitin and cellulose [18,21]. Their structures, occurring in all vertebrates, have been thoroughly described in the literature [21,22,23,24]. Keratins, synthesized in epithelial cells, are an integral part of the epidermis and its appendages, including hair, wool, feathers, nails, horns, hooves and cuticles [22]. Keratins form solid structures, held by disulfide bonds formed between the thiol groups (–SH) of cysteine amino acid residues, hydrogen bonds and hydrophobic interactions [21,24]. The multi-level structure and a high number of cross-linkages between various types of keratins results in high resistance to mechanical, chemical and physical factors [23]. They are characterized based on their secondary structures (mainly α-helixes and β-sheets), sulphur content (soft and hard), amino acid composition (basic, acidic or neutral), molecular weight and a source of origin [21,22,23,24].

Based on their secondary structure, three main types of keratin have been recognized–α-keratin (with α-helices), β-keratin (with β-sheets) and γ-keratins (Table 1) [21,24,25]. The keratin-associated proteins (KAPs) that create a matrix between keratin fibrils are occasionally distinguished as a separate group from γ-keratins [24,26].

However, the content of α- and β-keratin, as well as the exact amino acid composition, can vary depending on the origin of the biomass, and also based on the material’s role and its place of growth [21,23,24]. The feathers of the outer layer, called cover feathers, usually consist mostly of the harder β-keratin. The keratins of bird feathers contain about 2.5 times more cystine residues formed as a result of joining two cysteine residues with a disulfide bridge than the keratins of nails, horns or the epidermis [21,22,24].

Based on the sulfur content derived from cysteine residues, keratins can be also divided into soft and hard keratins [21,24]. Soft keratins, usually containing up to 1% of sulfur (or up to 10% of cysteine residues) in their composition, are found in the cytoskeleton materials of epithelial tissues, e.g., in the intermediate filaments. Due to the lower content of cysteine and thus disulfide bridges, soft keratins are more flexible than the hard keratins. They play an important role in cell division, transmission of signals in and out of cells and its spatial organization [22,27,28]. Hard keratins, an essential component of hair, wool, nails, feathers, beaks and claws, can contain from 1% to 5% of sulfur (10–14% cysteine residues). Their role is to protect against mechanical disruptions, predators and abiotic factors, such as low or high temperature or high doses of radiation [21,22,24]. Keratins are hygroscopic, but insoluble in water, weak acids, alkalis and most organic solvents [29,30].

## 3. Conventional Keratin Degradation

The main physicochemical methods of keratin treatment are solubilization in organic solvents or ionic liquids, hydrothermal treatment, acid or alkaline hydrolysis, oxidation or reduction in disulfide bridges by the addition of chemicals, and degradation of hydrogen bonds, e.g., by urea [23,24,31,32,33]. The choice of the treatment method has a significant impact on the composition of the extracted keratin and keratin hydrolysate and largely depends on the intended future use of the final product [30,31,34,35,36]. A comparison of various physicochemical treatment methods with their advantages and disadvantages are presented in Table 2.

Although physicochemical methods are relatively versatile and efficient, they usually lead to a significant modification of the amino acid composition of the extracted keratin or the hydrolysate. The final product contains free amino acids and peptides; however, depending on the method used, also specific amino acid derivatives or their stereoisomers [24,37]. Such products usually require further, often costly, isolation and purification. Therefore, the traditional waste management (e.g., incineration or landfilling) as well as physicochemical treatments are regarded as unsustainable, as they contribute to space shortages, increased usage of concentrated and hazardous chemicals, eutrophication, elevated emissions of greenhouse gases, high energy consumption and the necessity of potentially hazardous waste hydrolysate management. Therefore, degradation with the use of keratinolytic microorganisms and their enzymes is seen as a potentially beneficial alternative to conventional management methods [8,24,33].

## 4. Microbial and Enzymatic Biodegradation

Keratinases (E.C. 3.4.21/E.C. 3.4.24/E.C. 3.4.99.11) are a group of proteolytic enzymes that catalyze the breakdown of peptide bonds in keratins [38]. Even though keratins are one of the most abundant biomass produced, they do not accumulate significantly in ecologically stable environments [39]. This, along with an extensive amount of already classified keratin-degrading microbial strains shows that the presence of keratinases or keratin-degrading proteases is not limited to very specialized ecological niches, but rather common throughout the globe. Keratinases are synthesized by many bacteria and fungi [39]. Up to date, the most important producers of keratinolytic enzymes are bacteria of the genus *Bacillus*, including *B. subtilis* and *B. licheniformis*. Other bacterial producers belong to *Micrococcus* sp., actinomycetes (*Streptomyces* and *Thermoactinomycetes* genera), fungal *Aspergillus*, *Alternaria*, *Penicillium* and *Chrysosporium* genera and many more [18,38]. Keratinases are also produced by fungal pathogens, such as *Trichophyton rubrum*, *T. mentagrophytes*, *Microsporum gypseum* and *Epidermophyton floccosum* [40]. Reportedly, black carpet beetle (*Attagenus unicolor*) might be a possible producer of keratinolytic enzymes, as its transcriptome holds many gene sequences homologous to known microbial keratinases, and was altered when insects have been fed with poultry feathers [41].

Every year, new microorganisms and microbial strains with keratin-degrading abilities are isolated from various environments, including feather waste landfills, skin surface, soil and water bodies near slaughterhouses, industrial wastewaters and organisms such as insects and spiders [39,42].

### 4.1. Keratinases Properties

Keratinolytic enzymes have various optimal temperature and pH ranges [18,43]. Keratinase from *Brevibacterium luteolum* is characterized by optimal temperature (T_opt_) of 30 °C [44], whereas the enzyme from a thermophilic bacterium *Fervidobacterium islandicum* AW-1 [45] exhibits T_opt_ of 100 °C. Even though most keratinases have an optimum activity at or above pH 7, *Trichophyton mentagrophytes* produces the keratinolytic enzyme with the highest activity in slightly acidic pH 5.5 [46]. On the other hand, the highly-alkaline bacterial keratinase from *Brevibacillus* sp. AS-S10-11 has been characterized by optimal pH (pH_opt_) of 12.5 [47]. Molecular weight of microbial keratinolytic enzymes can range from 20 [48] to even 240 kDa [18,49]. Recently isolated keratinolytic enzymes and their biochemical properties are shown in Table 3.

Most keratinases are typically synthesized in the late exponential and/or in stationary phase and it is most probably related to the time needed for metabolism adaptation to the lack of nutrients in a given environment. As keratinolytic enzymes are regarded as inductive biocatalysts, their synthesis is carried out mainly in response to a keratinous substrate presence [18,44,46,72,73,74,75,76]. Reportedly, other protein components of microbiological media, such as tryptone, casein or yeast extract are, however, less effective kertinases inducers [18,19,66,77]. Keratinolytic enzymes exhibit a broad substrate specificity, as they can catalyze the breakdown of hair, feathers, wool, horns, hooves, scales keratins and even other fiber-forming proteins, such as collagen and elastin [18,19,66]. The degradation of non-fibrous proteins, such as casein and albumin, is also well documented, and is often used in microbial isolation and screening procedures [19,39,78]. Many keratinolytic enzymes are active against synthetic peptides such as Z-Ala-Ala-Leu-pNA, Z-Gly-Gly-Leu-pNA, Z-Gly-Pro-Cit-pNA and Suc-Ala-Ala-Pro-Phe-pNA, corresponding to the subtilisin substrate specificity [22]. Enzymatic activity of keratinases is diverse and generally falls in the range of 10 to 18,000 U/mL [72]. However, it is worth noting that methods of keratinase activity assay are a subject of greater discussion, as there are many available [75] (see Table 4) and each is characterized by different advantages and disadvantages with little possibility for comparison of final results [19,74,79]. 

**Figure 1:**
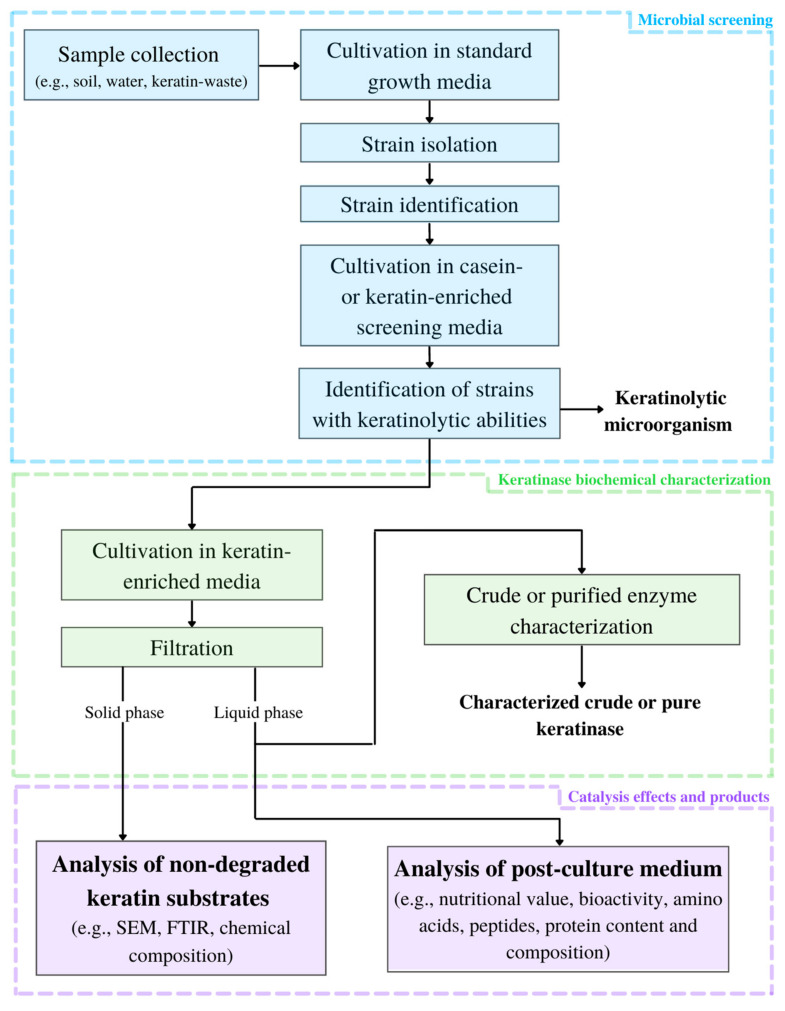
Framework for keratinolytic enzyme characterization (SEM—scanning electron microscope; FTIR—Fourier transform infrared spectroscopy).

Framework for keratinolytic enzyme characterization is shown at Figure 1.

### 4.2. Catalytic Mechanism

Keratins, due to their compact structure (with compromised access to specific, recognized peptide bonds and residues) and insolubility, are a challenging substrate to hydrolyze for most common proteases. Even though many keratinolytic enzymes have been isolated over the years, the precise mechanism of keratin biodegradation is not fully understood [62,77]. A vast majority of keratinases are extracellular enzymes [56,59,60,62]. However, intracellular enzymes with keratinolytic activity from *B. licheniformis* RGI and *Trichophyton gallinae* were also reported [95,96]. Keratin biodegradation is thought to be composed of enzyme’s adsorption to the macromolecule’s surface (possibly by electrostatic and hydrophobic interactions) followed by the proper catalytic action. This action can be further divided into reduction in disulfide bonds, also called sulfitolysis, and disruption of the peptide chain (proteolysis). Based on proteolysis catalytic strategy, the majority of keratinases are representatives of serine- or metalloproteases. Sulfitolysis is thought to happen only in the presence of reducing agents, such as sodium sulfite, β-mercaptoethanol, dithiothreitol (DTT), thioglycolic acid, glutathione or cysteine. Enzymatic sulfitolysis is also possible [62,77]. It is also suggested that proteolytic enzymes might be involved in a catalytic synergy with disulfide reductases (oxidoreductases) acting as another group of disulfide-bond-reducing agents and a facilitators of keratin biodegradation [42,62,77]. Nevertheless, many keratinases from the *Bacillus* genus have been described as the sole catalysts of keratin degradation [19,97]. Therefore it is not ruled out that various microorganisms developed different mechanisms of keratin degradation, using self-produced reducing agents, membrane-associated, extracellular keratinolytic enzymes or their combinations, with intracellular proteases further degrading proteins and peptides after they are transported into cells [42,77]. However, it is not proven whether such cross-class enzymatic synergy is more common for keratin-degrading microorganisms or a highly specific mechanism developed by the thermophilic bacterium, *Fervidobacterium islandicum* AW-1 [77], as a response to extreme environmental conditions. Moreover, it was stated that a group of enzymes, called lytic polysaccharide monooxygenases (LPMOs), associated for some time with cellulose, chitin, starch and hemicellulose polymer degradation, may also contribute to the disruption of α- and β-keratins [98,99,100].

### 4.3. Keratinases Immobilization Methods

Though enzymes are a group of catalysts often praised by their effectiveness, selectiveness and sustainability, their application in many industrial scale processes has been limited due to their relatively high production costs, challenging recovery and difficulties with maintaining sufficient stability in catalysis conditions. To mitigate such drawbacks, many strategies, including enzyme immobilization methods, have been developed [101,102,103]. The main techniques used for biocatalysts immobilization on/with solid supporting materials are (1) adsorption, (2) entrapment, (3) encapsulation, (4) covalent binding and (5) cross-linking, and their combinations [101,102,103,104,105,106,107,108,109,110]. Specific immobilization conditions and choice of suitable matrix material have to be optimized and tailored to the given enzyme and its targeted application. Keratinolytic enzymes have also been subjected to immobilization methods employing various materials, e.g., charcoal, glass beads, silica gel, cellulose, chitosan, chitin and alginates [101,102,104].

Commercially available recombinant keratinase from *B. licheniformis* was successfully immobilized with chitosan-glutaraldehyde (chitosan-E) and chitosan-cyclodextrin-glutaraldehyde (chitosan-β-CD-E) beads with high immobilization yield (90 and 93%, respectively), resulting in increased storage stability and allowing for biocatalyst reuse in wool conditioning processes [103]. Calcium alginate gel beads has been used to entrap *B. licheniformis* LMUB05 keratinase. Immobilized enzyme, implemented in generation of keratin-derived feed, exhibited increased pH tolerance and thermal stability in comparison with the crude enzyme [105]. Another recombinant keratinase KMT-wt from thermophilic *Meiothermus taiwanensis* WR-220 was immobilized on modified bagasse cellulose and utilized in wastewater treatment [106]. Immobilizing *B. subtilis* RM-01 keratinase with poly(vinyl alcohol)-assisted silver-nanoparticles (PVA-AgNPs) resulted in increase in specific activity, thermostability and storage stability of the biocatalyst. Additionally, due to immobilization the enzyme exhibited enhanced antibacterial and dehairing activity [107].

Biocatalyst immobilization with polyethylene glycol-assisted (PEG) iron oxide (Fe_3_O_4_) magnetic nanoparticles (MNPs) was proven to facilitate recovery of immobilized keratinases of *B. subtilis* [108] and *B. licheniformis* AS-S24-I using magnets [109]. Interestingly genetic engineering can also be introduced in keratinolytic enzymes immobilization strategies. Recombinant keratinase from *B. licheniformis* PWD-1 was expressed as keratinase-streptavidin fusion protein resulting in affinity of fusion biocatalyst to a biotinylated materials. Moreover, such immobilization methods resulted in an increase in thermostability and improvement of the pH tolerance of the enzyme [110].

### 4.4. Genetic Engineering of Keratinolytic Strains and Enzymes

Pressure for agro-industries to produce more food for continuously growing human population is causing an increased generation of agricultural waste, including keratin-based waste [111]. Keratinases have many potential applications (described below) in various industries; however, their production from non-enhanced native hosts is generally regarded as insufficient to meet the large demand of the global market [112]. Moreover, many of efficiently keratin-degrading microorganisms and enzymes are thermophilic in nature [59,60,77,113,114], which might pose a threat to their economic and environmental sustainability. Several genetic engineering strategies have been developed and tested to (1) increase the amounts of secreted enzymes, (2) to facilitate the enzymes production, (3) to improve their biochemical properties and activity as well as (h4) to alter the substrate specificity [60,115,116,117,118,119,120,121,122,123,124,125].

Reportedly, a production of keratinolytic enzymes is significantly correlated with a composition of culturing medium, resulting in highest secretion at the relatively low keratin-substrate concentrations of about 1–5% (*w*/*v*). Further increase often decrease amounts of secreted enzymes. However, such determination is generally strain-specific and has to be examined with other culturing conditions [47,61,63,66,126,127,128]. To overcome this obstruction and to improve better cost-effectiveness of keratinase production, enzyme overexpression in the native host can be performed, using vector-integrated enzyme genes (possible to control thanks to plasmid construction), which can increase biocatalyst production even up to 5–6 times [115,116]. Similarly, random or specific mutagenesis of native strains, caused, e.g., by UV-radiation or ethyl methanesulfonate, can result in obtaining strains with better keratinolytic abilities [117,118]. Another strain engineering strategy is to produce metabolically impaired native strain, unable to produce certain intra- and extracellular proteases, signal peptides and RNases [125]. Such approach can result in obtaining hosts more suitable for secretion of heterologous, as well as homologous proteins [72,129]. Keratinolytic enzymes can be also cloned from newly isolated, often demanding, strains and expressed in a well-known and widely-used heterologous bacterial, e.g., *Escherichia coli* and *B. subtilis*, and fungal hosts, such as *Pichia pastoris* [18,72,130]. Heterologous expression of *B. lichenifomis* PWD-1 keratinase (KerA) have also been reported in insect cells of *Spodoptera frugiperda* (*Sf9*) [131]. 

Many molecular strategies, dedicated for each heterologous host, have been developed over the years, for further improvement of keratinases production, biochemical property enhancement and down-stream processes, including adjustment of available vectors to a given host, choice of suitable promoter, modification of enzyme propeptide sequences and chromosomal integration [72,112,129]. Examples of keratinolytic strains, enzyme engineering strategies and their outcomes are presented in Table 5. 

## 5. Application

Keratinolytic enzymes and their microbial producers have been already been found to be applicable in several industrial sectors. Some microbial keratinases, especially from *Bacillus* spp., are available as purified enzymes, enzyme preparations or as bioactive ingredients in commercial products. Examples of some commercially available keratinolytic enzymes and their biotechnological applications are listed in Table 6 [17,19,39,132].

**Figure 2:**
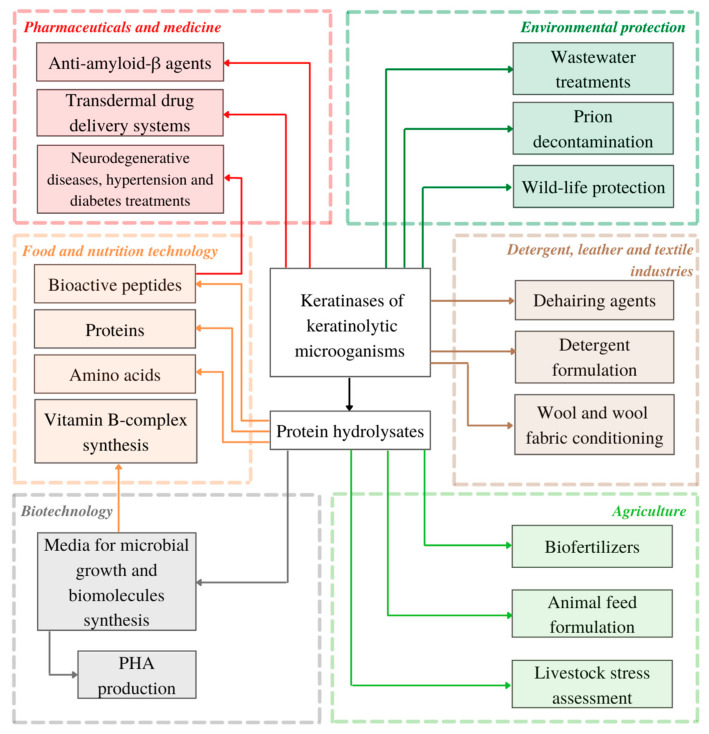
Present and potential applications of keratinolytic microorganisms and keratinases reviewed in this article (PHA—polyhydroxyalkanoates).

In many studies, when dealing with keratinous waste management, keratin substrate is transformed into protein-, peptide- and amino acid-rich hydrolysate as the main product of enzymatic or full-cell hydrolysis [34,37,97,133,134,135]. As keratin waste streams are constantly growing, keratinases and keratin-degrading microbes, can be utilized not only as waste decontamination agent, but also as novel tools for more sustainably obtained products with added value, ranging from animal feed, biomaterials and highly efficient dehairing agents to pharmaceuticals and bioactive peptides of therapeutic importance (see Figure 2).

### 5.1. Nutrition and Food Technology

#### Bioactive Peptides

Conventional raw substrates and food are nowadays regarded not only as a source of necessary nutrients crucial for maintaining organism’s homeostasis, but also as a source of industrially important bioactive molecules [136]. Bioactive peptides are fragments of the amino acid sequences of native proteins, which remain inactive in their precursors. They can also be synthesized by single-cell bacteria [137]. When released, they can act as regulators of numerous physiological processes and endocrine (oxytocin, adrenocorticotropic hormone, and calcitonin), immune, circulatory, nervous and digestive systems [136,137,138]. Type of biological activity, displayed by biopeptides, is main determinant of their division (see Table 7) [136]. Many bioactive peptides can exhibit antioxidant, antimicrobial (gramicidins, polymyxins, bacitracins), anti-amnesic or even opioid-like activities. Some can lower blood pressure (angiotensin-I converting enzyme (ACE) inhibitors; dipeptidyl peptidase-IV (DPP-IV) inhibitors; Ile-Tyr, Lys-Trp, Ile-Trp dipeptides), whereas others can bind metal ions or shape sensory properties of food (nisin, aspartame, lipopeptides). They are currently used in the generation of functional products and nutritional formulations [136,137]. The bioactive peptides are also attractive molecules for pharmaceutical and cosmetic applications [136,137,138]. It is estimated that the peptide-based product market value exceeds USD 40 billion [139].

Technologies for bioactive peptides production include (1) natural source extraction, (2) genetic engineering, (3) cell-free expression systems, (4) transgenic animals and plants and (5) chemical and enzymatic synthesis [137,138,140,141,142,143,144].

Event though, most of the bioactive peptides were identified in food and food by-products [136,139], waste biomass can also become a source of molecules with bioactive properties [34,37,133,142,145]. Extraction from natural sources is a commonly used strategy for releasing and obtaining peptides from food proteins, based on (a) in vitro enzymatic hydrolysis, (b) cultivation of protein-degrading microorganisms (fermentation) and (c) animal digestive enzymes hydrolysis [136,138]. For in vitro food-protein hydrolysis, proteases with broad substrate specificity, from plant (ficin, bromelain, papain), animal (pepsin, chymotrypsin, trypsin) and microbial (proteinase K, collagenase, subtilisin A) sources, are needed [136]. As the representatives of proteolytic enzymes, keratinases can be potentially used for bioactive peptides production via enzymatic hydrolysis of keratins and other proteins, or by full cell fermentation of microbial media supplemented with poultry feather and non-keratin plant proteins [68,142,145].

Several studies, exploring the possibility of obtaining bioactive hydrolysates and peptides from keratin waste have been conducted. Three previously evaluated *Bacillus* strains of keratinolytic activities, CL18, CL33A and CL14, were used for full-cell bioconversion of chicken feathers into protein hydrolysates [133] via submerged cultivation in feather broth (FB) medium (0.5 g/L NaCl, 0.3 g/L K_2_HPO_4_, and 0.4 g/L KH_2_PO_4_) with whole chicken feathers (10, 20, 30, 40 and 50 g/L). Fermentation was conducted at 30 °C, pH 7 and with 125 rpm for at least 13 days (different for each strain). Obtained hydrolysates were tested for DPPH and ABTS radical scavenging activities, Fe^2+^-chelating ability, as well as for inhibition of dipeptidyl peptidase IV (DPP IV) and angiotensin I-converting enzyme (ACE). Out of all three strains, *Bacillus* sp. CL18 degraded feathers most effectively, resulting in degradation of 98.9% of feathers after 7 days of cultivation in FB medium supplemented with 10% of feathers (FB10), followed by *Bacillus* sp. CL33A (56.2% after 9 days) and *Bacillus* sp. CL14 (71.4% after 13 days). All hydrolysates showed a wide range of bioactive activities in all tested concentrations of chicken feathers in the FB. Maximum of 95.7% of DPP IV and 89.7% of ACE inhibition was observed in feather hydrolysate after 5-day cultivation of *Bacillus* sp. CL18 in FB40, as well as significant antioxidant and reducing activities. Therefore protein hydrolysates after *Bacillus* sp. CL18 full-cell fermentation, might be potentially a rich source of bioactive peptides of nutritional and pharmaceutical importance [133]. 

In another study, keratinase from *Bacillus* sp. CL18 was partially purified and applied for conversion of various protein substrates into bioactive hydrolysates [142] at optimal conditions (55 °C; pH 8.0; 5 mM Ca^2+^). From all tested substrates, casein was the most preferable for hydrolysis (100% relative activity), followed by soy protein isolate (83%), albumin (26.8%), feather meal (19.4%) and non-pretreated keratinous substrates such as human nails (7.6%), chicken nails (5.2%), chicken feathers (4.5%) and human hair 2.8% [142]. Soy protein isolate by itself had antioxidant properties; however, after 4 h of keratinolytic hydrolysis, the ability to scavenge the ABTS radicals increased from around 17.5% to almost 69% and from 27% to around 40% of DDPH radicals. Moreover, increased inhibition of ACE (from 10% to maximum 89.1%) and DPP IV (form 4.7% to maximum 34.8%) were also observed as another result of soy proteins hydrolysis with partially purified keratinase from *Bacillus* sp. CL18 [142].

Feather hydrolysates were also obtained by full-cell fermentation of *Bacillus cytotoxicus* LT-1 and *B. cytotoxicus* Oll-15 [68]. Minimal mineral media (2.48 g/L K_2_HPO_4_, 0.49 g/L NaH_2_PO_4_, 0.01 g/L MgCl_2_, 0.02 g/L FeCl_3_, 0.1 mg/L CaCl_2_ and 0.013 g/L ZnCl_2_; pH 6.0) with 10 g/L of chicken feathers and given bacterium, were incubated 48 h at 50 °C with 150 rpm. Enzyme production of strain Oll-15 was the highest after 24 h of cultivation (14.4 U/mL), whereas strain LT-1 reached maximum production (16.6 U/mL) after 33 h. Hydrolysate from strain Oll-15 exhibited slightly better reducing activity than ascorbic acid (used as a positive control), whereas LT-1 hydrolysate did not show any significant reducing properties. Both Oll-15 and LT-1 hydrolysates showed noticeable scavenging activity of 85 and 64%, respectively, suggesting the presence of bioactive peptides in post-culture media [68].

Recombinant keratinase KerZ1 from *B. licheniformis* BBE11-1, expressed in *B. subtilis* WB600 (with maximum activity of 426.6 kU/mL in 15-L bioreactor), was used for feather degradation in an optimized fermentation medium (10 g/L yeast extract, 20 g/L tryptone, 20 g/L sucrose, 3 g/L KH_2_PO_4_, 6 g/L Na_2_HPO_4_, and 0.3 g/L MgSO_4_) supplemented with disulfide-bonds reducing agent, 1% sodium sulfate. Such combination allowed for efficient degradation of poultry feathers at 60 °C and pH 7.0 within 12 h, which resulted in a release of a wide variety of amino acids (with relatively high content of Glu, Ala, Tyr, Phe, Leu and Lys) with maximal conversion rate of 56.6%, when 100 g/L of feather were used [145]. Beside amino acids, hydrolysate contained a mixture of short peptides (~1.3 kDa) with at least 12 of them exhibiting catalytic and antioxidant activities. However, the exact sequences and properties of those bioactive peptides have yet to be determined [145]. 

Even though production processes of waste-derived bioactive hydrolysates and peptides require further research, especially in terms of product purity, stability and safety, with optimized production methods and dedicated delivery systems [139], they may become a promising and more sustainable alternative to food-derived [136], GMO/GMM-derived and enzymatically or chemically synthesized [137] peptides for human and animal consumption. 

### 5.2. Agriculture

Agriculture is one of the most significant sectors of global economy. However, animal husbandry, especially industrial meat and dairy production, is greatly contributing to climate change by the native habitat uptake, biodiversity loss, generation of hazardous waste, eutrophication and significant green-house gases emissions. As current and future challenges, connected with satisfying the demands of growing human population and rise of environmental issues are more apparent, more sustainable methods for food, feed and nutrient generation are of great interest [7,8,12,146].

#### 5.2.1. Biofertilizers and Plant Biostimulants

Despite being a valuable source of proteins, peptides and amino acids (both essential and non-essential), keratin hydrolysates, including feather-derived ones, are also regarded as a good source of nitrogen (N), phosphorus (P), and other minerals, e.g., potassium (K), calcium (Ca) and magnesium (Mg) [146]. As use of synthetic fertilizers can negatively impact the environment, feather-derived hydrolysates are studied for their biofertilizing proposes. Biologically obtained keratin hydrolysates generated during full-cell hydrolysis are of notable interest, mainly due to the possibility of additional plant biostimulants synthesis by fungi or bacteria during fermentation period (see Table 8). Plant biostimulants (PBs) are a wide group of molecules, often microbial metabolites, such as indole-3-acetic acid (IAA) with beneficial effects on plants growth and safety, including uptake of nutrients, improvement of stress tolerance and nutritional value of crops, as well as antimicrobial activity [24]. Moreover, utilization of feather waste biofertilizers can also improve quality of soil by increasing water retention and inorganic phosphorus solubilization [146].

Studies showed that biofertilizer produced by full-cell hydrolysis of feather waste using *Bacillus aerius* NSMk2 and minimal salt medium with chicken feather as sole carbon source (3 days, at 35 °C, pH 7.5), stimulated germination of seeds and further growth of mung beans (*Vigna radiata*) when supplemented to the soil [147]. Similar observations were noted with activation of proton pump when tomato (*Solanum lycopersicum*) seedlings were cultivated in a soil enriched with keratin-based fertilizers, obtained by *Trichoderma asperellum* T50 and *T. atroviride* full-cell hydrolysis of wool or feathers conducted on minimal medium (0.5 g/L NaH_2_PO_4_, 0.5 g/L KH_2_PO_4_, 0.016 g/L FeCl_3_, 0.1 g/L CaCl_2_, 0.01 g/L MgCl_2_) with 1% *w*/*w* of keratin substrates for 21 days at around 26 °C and pH 7.0 [148].

Many amino acids can act as biostimulants, as they regulate normal and stress-induced metabolism of plants [149]. IAA is a phytohormone synthesized by both plants and keratinolytic microbes from tryptophan; therefore, the presence of this amino acid or IAA itself in biofertilizer can greatly contribute to faster and more efficient plant development. However, taking into consideration various chemical composition of keratin-rich animal waste can significantly affect final content and availability of certain proteins, peptides and amino acids in keratin hydrolysate, tailored approach for each application might be required [20,132].

#### 5.2.2. Animal Feed

The Life Cycle Assessment (LCA) studies focused on environmental impact (EI) of meat and dairy industries in Europe and other regions have shown that animal feed production and supply are two major contributors of global warming, eutrophication and acidification potential [12,150,151]. Depending on the origin and supply chain of feed ingredients, land use change and pesticides utilization have become other factors of great concern. This is especially significant for protein sources, such as soy and its derivatives, mostly imported from South America [12]. Moreover, the heavy dependence of the poultry sector on soy creates a non-negligible food security risk for European countries. Research focused on transforming the waste streams such as chicken feather or animal hair into novel sources of nutrients seems to be especially valid in the EU, as up to 77% of required protein for feed and food supplies is imported from the outside of the EU member states [152]. As poultry feathers are among the most widely distributed keratin-rich industrial waste globally, attempts to converse it to novel and potentially beneficial protein products are not surprising. Examples of keratinolytic microorganisms or enzymes utilized for bioconversion of keratin waste into animal feed are presented in Table 9.

As study shows, steam hydrolyzed feather meal can be a more sustainable substitute for a fish meal in aquaculture [150]. However, little is yet known about the LCA and potential EI of feather-waste-derived feed hydrolysates obtained by microbial or enzymatic degradation. Reportedly, supplementing animal feed with keratinases improves its digestibility, as well as promoting microbiota growth and immune response in livestock [72]. Therefore, bioconversion of keratin waste into animal feed using whole cells and/or keratinolytic enzymes, might be a promising approach for better sustainability of the meat industry, as feed or protein digestibility positively impact the feed conversion ratio, which is strongly correlated with the animal feed environmental impact [150,151].

Microbial (bacterial, yeast, microalgal), insect and waste-derived proteins are amongst the top options regarded as more sustainable sources of nutrients than soy and its derivatives; therefore, their implementation needs further exploration and research [151]. However, some of microbial strains used for a small scale full-cell hydrolysis of keratin-rich substrates for enzyme, peptide and amino acid production, are known human and animal pathogens [84]. The presence of endotoxins and risk of the spread of infectious diseases are main concerns for the usage of some native keratinolytic microbes; therefore, their direct applicability on a larger scale is compromised [25]. 

#### 5.2.3. Livestock Stress Assessment 

Unsuitable and improper breeding conditions in large-scale industrial meat plants are among the factors that can affect the levels of steroid stress hormones, glucocorticoids (GCCs), in animal bloodstreams [154]. Increased blood concentrations of GCCs can lead to suppression of growth and reproduction, as well as overall immunodeficiency. Therefore, stress management should be a necessity for improving and maintaining animals’ well-being. As GCCs are secreted in response to many, potentially stressful, environmental factors, their levels can be used for an overall assessment of population stress, an assessment of long-term response to a newly introduced factor or for an effective control of stress-mitigating solutions. The main GCC checked in birds, including avian livestock, is called corticosterone (CORT) [155]. However, current methods of CORT indexing are mostly based on blood samples, which collecting is an event stressful itself and have to be repeated multiple times for long-term stress assessment. The CORT levels in feather tissue corresponds with its blood concentration during its period of growth [155]. Moreover, GCCs deposited in feathers are stable, thus samples do not need refrigeration or immediate extraction and hormone measurement [156]. Due to known resistance of keratins to proteolysis with conventional enzymes, keratinases have been used as promising agents for enhanced digestion of feathers and improved extraction of CORT from samples [154]. For that purpose, commercial keratinase from *B. licheniformis* (Cibenza IND900, Novus International, Inc., St. Charles, MO, USA) was applied as the enzyme-digesting keratin sample. Serial dilutions (with PBS buffer, pH 9.0) were performed to specify which enzyme concentration will be the most suitable for further extraction of radiolabeled GCCs. Feather samples have been incubated with enzymatic solutions of pH 9.0 at 45 °C for 5 days and observed [154]. From all tested concentrations, only undiluted keratinase solution (1:30) liquefied feathers to the point suitable for solid-phase extraction (SPE) techniques. Implementation of enzymatic digestion allowed for consistent and repetitive hormone extraction and development of a new accurate and reliable detection of CCS at very low concentrations (pg/mL). As study suggest, presented CORT assay protocol may be extended to different types of animal samples, as well as to other GCCs and biomolecules in various taxa [154].

### 5.3. Biotechnology

#### Microbial Media

The bacterium *Pseudomonas* sp. P5 has been utilized for the valorization of poultry feathers as a substrate for the production of protein hydrolysates with potential application as microbial media [37]. An isolated strain with the highest proteolytic and keratinolytic activity was cultured on mineral medium supplemented with raw wet feathers (form 30 g/L to 90 g/L). After 5 days of incubation, feather substrate hydrolysis rate varied from 70 to up to 93% and was correlated with initial concentration of feather supplement (highest rate observed for lowest feather concentration) [37]. However, with higher levels of keratin-substrate (70 and 90 g/L), the weight of bacterial biomass, the amount of extracellular enzyme and its activity increased, resulting in the maximum keratinolytic activity of 36.2 ± 8.4 U/mL (for 70 g/L). In this study, the final content of peptides and amino acids in the medium after full cell fermentation by *Pseudomonas* sp. P5 was compared with enzymatic (partially purified keratinase from the same bacterium; temp. 50 °C, pH 7.5, 24−48 h, with 1–2 U per 1 mg of dry feathers), weak alkaline hydrolysis (0.6% KOH, 70 °C, 24 h) and control conditions, with two same feather concentrations of 70 and 90 g/L. As the result, hydrolysate after full-cell fermentation contained significantly lower amounts of amino acids, 301.2 mg/L (90 g/L of wet feather) and 274.8 mg/L (70 g/L of wet feather), than the enzymatic hydrolysate, 1047.6 mg/L (90 g/L of wet feather) and 1090.7 mg/L (70 g/L of wet feather). However, peptides concentration was slightly higher in hydrolysate after full-cell fermentation (6.2 and 4.6 g/L compared with 3.2 and 3.3 g/L in enzymatic hydrolysate) [37]. Alkaline hydrolysis resulted in the highest number of peptides (17.2 g/L and 14.3 g/L) of all types of feather treatments, but lower concentrations of amino acids (326.9 mg/L and 377.8 mg/L) than in the enzymatic hydrolysate. Amino acid profiles were tested using ultra-high-performance liquid chromatography (UHPLC). Essential amino acids accounted for nearly 54% of all amino acids in post-culture liquid after enzymatic treatment, 44% after direct fermentation with *Pseudomonas* sp. P5 cells and only 12.5% after alkaline hydrolysis [37]. 

Obtained hydrolysates were further tested as culture medium for *E. coli*, a well-known and widely used model microorganism in biotechnology. Enzymatic and alkaline hydrolysates tested positive with no additional ingredients and operations (except pH adjustment) needed for satisfactory bacterial growth. The development of non-pretreated keratin substrate bioconversion (excluding the need for washing, milling or grounding) could positively affect cost of medium preparation; therefore, this increases the feasibility and applicability of the biotechnological process [37]. Though with time such hydrolysates can become a less expensive alternative for peptones in popular microbial media, more research is still required as commercially available protein ingredients are produced in well-optimized processes with product being filtrated for the removal of potential inhibitors, toxins and molecules of high molecular mass [37].

Keratin waste hydrolysates produced by enzymatic in vitro proteolysis or full cell fermentation of keratin substrates, can be also utilized for synthesis of organic micronutrients with crucial metabolic activities, such as vitamin B [25]. As the study shows, crude keratinase from newly isolated *Bacillus thuringiens* is MT1 was successfully used for preparation of amino-acid-rich hydrolysate from donkey hair [25]. The release of amino acid was obtained by incubation of previously washed and dried hair (0.5 g) with diluted crude enzyme (250 mL; glycine/NaOH buffer; final activity of 20 U/mL) at 50 °C, pH 9 and 100 rpm for 24 h. After incubation, the concentrations of amino acids were tested and their different levels were supplemented to YPD broth as precursors of vitamin B-complex (B1, B2 and B12) production by *Sacharomyces cerevisiae* (ATCC 64712). As a result, donkey hair hydrolysate was composed of 16 amino acids, half of them being essential amino acids (Met, Phe, Thr, Val, Leu, Ile, Lys and His) and other 8 the non-essential ones (Glu, Asp, Pro, Ala, Arg, Gly, Ser and Tyr) with concentrations ranging from 13.5 (Ile) to 32.3 (Lys) µg/mL and from 7.0 (Tyr) to 143.7 (Glu) µg/mL, respectively [25]. Analysis of enzyme-free post-incubation liquid revealed no free amino acids. Several concentrations of hair hydrolysate (from 0.0 to 2.5% of total amino acid) were used as supplement in *S. cerevisiae* culture for vitamin B-complex synthesis. The highest amounts of all three vitamins, B1, B2 and B12, were obtained with 1.5% of free amino acid solution at concentrations of 0.04, 0.06 and 0.06 mg/mL, respectively. In control culture (without hair hydrolysate supplementation), the presence of a vitamin B complex was not detected [25]. The bioconversion of donkey hair into a production medium for vitamins synthesis, though requiring a further optimization, seems to be a promising new method of keratin waste management. As the study suggests, other keratin-rich substrates can also be valorized for vitamin B complex production, as they often have a different chemical and protein structure, which may affect the final content and variety of free amino acids in potential production medium, as well as the amount of particular vitamins synthesized within the B complex [25].

### 5.4. Pharmaceuticals and Medicine

#### 5.4.1. Transdermal Drug Delivery Systems 

Many keratin-degrading microbes, especially fungal, are also human pathogens, often causing long-lasting and troublesome skin infections. Those microorganisms produce keratinolytic enzymes in order to infect and penetrate keratin-based tissues. However, such enzymatic virulence factors may also find novel application in transdermal drug delivery systems, where they could act as effectiveness-enhancing reagent for better and deeper delivery of antibiotics and other molecules for deep skin infections treatment [63]. Keratinase from *Bacillus cereus* was isolated from samples of farm soils in Egypt, biochemically characterized and tested in the transdermal drug delivery system (TDDS), composed of keratinolytic enzyme and fucidic acid cream. The effectiveness of the proposed TDDS was studied on mice, subcutaneously infected with 50 µL of *Staphylococcus aureus* ATCC 25,729 (2 × 10^9^ CFU/mL) suspension—an equivalent to 1 × 10^8^ CFU. Each group was observed, with complete healing periods being recorded. After that time, viable counts of *S. aureus* in skin sample homogenizates were examined using the mannitol salt agar plate cultivation method. As the results show, keratinase exhibited a relatively large thermal stability range by retaining up to 90% of primary activity after 16 h of incubation in temperatures between 4 °C and 50 °C. The enzyme maintained high activity after incubation for 16 h at pH 7.0, and about 90% after 2 h at pH 9.0. However, keratinase was quite sensitive towards more acidic pH. Implementation of keratinase into standard treatment of fucidic acid cream resulted in significant reduction in the time needed for complete healing from 7 days (for group treated only with fucidic acid) to only 5 days, with a notable decline in *S. aureus* viable count of about 2 log cycles on the fourth day of infection treatment [63].

Keratinolytic enzymes, acting as an additive for improved delivery of therapeutics and antibiotics, e.g., for deep skin bacterial infection treatments, opens yet another way for chicken feather waste management and rational keratinases application in novel industry. 

#### 5.4.2. Anti-Amyloid- β Agents

Amyloids are misfolded forms of regular proteins, that can interact with themselves and other agents, developing insoluble fibrous aggregates resistant to proteolysis. Deposition of such aggregates is main or co-responsible reason behind hereditary lysozyme amyloidosis, Alzheimer’s disease (AD), Huntington’s disease, Parkinson’s disease, prion diseases and many others [157]. When toxic peptide particles, from 39 to 43 amino acid residues long, known as amyloid-β peptides (Aβ), are aggregated in extracellular space between nerve cells in brain tissue, they block synaptic transmissions, what results in degeneration of neurons and AD symptoms and progress [157]. Because keratinases seem to be specialized in degrading β-sheet-abundant recalcitrant substrates, typically not available for common proteases, research on their potential application in degradation of Aβs could lead to promising results and development of novel therapeutic methods amyloidosis-associated diseases [157]. 

Two bacterial keratinolytic enzymes Ker1 and Ker2, derived from *Amycolatopsis* sp. MBRL 40, have been tested for degradation of Aβ fibrils, synthesized from hen egg white lysozyme (HEWL) used as a model system. Aβ fibrous precipitates were suspended in 50 mM phosphate buffer (pH 7.0) and incubated with purified keratinases at different concentrations (50, 100, 125, 250 and 500 mg/mL) at 40 °C and for various catalysis time (0, 3, 6, 12, and 24 h). Presence of amyloidal structures were then tested by immunoblotting with anti-Aβ antibody [157]. As the result, Ker1 (in conc. of 125 mg/mL) completely degraded Aβ fibrils after 24 h at 40 °C. Digestion was later confirmed by no signals in immunoblot (untreated fibrils showed a positive signal). Ker2 was less effective than Ker1 with partial solubilization observed after 24 h. For complete degradation, time of the process needed to be extended to 7 days. Neutral phosphatidylcholine and cationic DOPE/DOTAP liposomes, loaded with Ker1, were synthesized to determine, whether such formations can also degrade Aβ fibrils. Similar to soluble Ker1, both types of proteoliposomes exhibited excellent Aβ-digesting abilities, confirmed by immunoblotting [157].

Keratinolytic biomolecules could become an interesting alternative to other, currently tested drugs for amyloidoses-associated diseases, that can cause unwanted side-effects, such as hepatic, renal and retinal toxicity, subacute meningoencephalitis, as well as injection-site reactions [157].

### 5.5. Biomaterials

#### PHA Production

Microbial polyhydroxyalkanoates (PHA) are a group of biopolyesters heavily studied for their physiochemical, biochemical and mechanical properties [158]. Their mechanical properties and degradability can be changed and adjusted by monomeric composition of synthesized polymers, by manipulating production process parameters, substrates, down-stream processing modification and selection of microbial production strains. PHA have found numerous applications in medicine, e.g., drug delivery and tissue engineering [159,160]. Products composed of PHA polymers and copolymers have been already applied in agriculture [161] and in the food industry as biodegradable and renewable packaging materials [162]. In biotechnology, PHA scaffolds can be used for enzyme immobilization [163]. They are synthesized by various microorganisms, such as *Cupriavidus necator* [164] and *Pseudomonas* spp. [165], as carbon and energy source, when nitrogen availability in environment is limited. Protective and stress-resisting functions of PHA were also described [166]. To meet petroleum-based polymer’s market prices with sustainability, many efforts have been put into developing PHA production processes based on agricultural and food waste [167]. Keratinolytic microorganisms have been also tied up with synergetic production of medium-chain-length polyhydroxyalkanoates (mcl-PHA) using chicken feathers as sole carbon source for *Pseudomonas putida* KT2440 growth medium [168]. Bacterial biomass obtained after separation of degraded feathers was resuspended in PHA production medium, supplemented with waste frying oil. As a result of 72 h cultivation, 61.4% wt of mcl-PHA per cell dry weight and 1.42 g/l^−1^ mcl-PHA productivity yields were obtained. Biosynthesized polymer was composed of C-6 (27.2 %mol) and C-8 (72.8 %mol) monomers. As the study suggests, such approach may not only be used as a sustainable method for mcl-PHA production (after further optimization), but also as an enzyme (keratinase present in post-culture filtrate of growth medium) and animal feed (partially hydrolyzed chicken feather) coproduction/cogeneration [168].

### 5.6. Detergent, Leather and Textile Industries

Market of industrial enzymes is estimated to reach worth of USD 6.2 billion in this decade with market demand, partially driven by worldwide implementation of sustainable development policies, rising 7% annually [38]. Since the 1960s, many alkaline proteases were recognized as beneficial detergent additives and found application in formulations for stain removal [69].

In order to be applicable in detergent, leather and textile industries (see Table 10), keratinases should be stable in the presence of commercial detergent formulations, typically enzyme-denaturing (e.g., SDS or CTAB) or non-denaturing detergents (e.g., Triton X-100, Tween-20, Tween-80), alkaline pH (up to pH 12.5) and heavy metals [126]. In such conditions, most enzymes are unable to maintain their activity; however, many keratinolytic enzymes, when exposed to extreme abiotic factors, exhibit either good stability or even show higher enzymatic activity [19].

In the leather industry, keratinolytic enzymes can be applied as dehairing agents thanks to (unlike in alkaline or sulfide treatments) exhibition of high substrate specificity towards hair-building keratins, thus allowing for proteolytic hair removal without damaging collagen-rich skin [91]. For example, the keratinase from *Bacillus* sp. RCM-SSR-102, known as KER102, had maximum observed activity at 50°C and pH 10.0 (retaining > 90% of activity in the range of pH 6.0 to 12.0). Moreover, KER102 could retain around 55% of its activity in highly saline environment of 3.4 M NaCl. Presence of Mg^2+^ and Ca^2+^ was stimulatory for the enzyme, whereas Fe^2+^, Pb^2+^, Ni^2+^, Cu^2+^, Zn^2+^, Mn^2+^ and Hg^2+^ ions had inhibitory effects. Both Tween-20 (1% *v/v*) and Tween-40 (1% *v*/*v*) have slightly increased enzyme activity, whereas 1% *v*/*v* of Triton X-100 have not changed it notably. KER102 showed K:C (keratinolytic to caseinolytic activity ratio) of 0.70 and 0.65 for feather and keratin azure, respectively. Such activity ratio determination is yet another method used for differentiating proteases from keratinases. Ratio exceeding 0.50 characterizes keratinolytic nature of a biocatalyst [97].

In the textile industry, keratinases may find applications mostly in pretreatments and conditioning of wool and woolen fabrics, as they are troublesome to process due to the felting shrinkage, compromised dyeing efficiency and degumming necessity [43]. Conventionally, excessive washing of the material leads to the felting and fibers entanglement, limiting fibers dyeing ability [82]. In addition, standard degumming process is regarded as hazardous to human health and environmentally problematic, thanks to utilization of concentrated chemicals, high process temperatures and generation of burdensome wastewater [43]. In result, keratinolytic enzymes might become a “green” alternative to conventional methods. Recombinant keratinase from *B. licheniformis* (500 U/mL) was incubated with wool fabric for 2 h, at 50 °C and pH 8.5 with addition 0.5 g/l non-ionic surfactant [103]. As the result, improvement of hydrophilicity and reduction in tensile strength was observed. Moreover, the enzyme was successfully immobilized with chitosan-E (chitosan + glutaraldehyde) and chitosan-β-CD-E (chitosan + cyclodextrin + glutaraldehyde) beads with high efficiency (immobilization yield of 90 and 93%, respectively), allowing for enzyme reusability and increased storage stability (assay with keratin azure), and thus potentially improving cost-effectiveness of enzymatic wool conditioning. However, enzymatic activity of immobilized keratinase towards wool and wool fabrics has yet to be determined [103].

Possibility of replacing environmentally unsustainable and hazardous chemicals with biodegradable enzymes in one of the highest polluting industries without loss of efficiency and generation of next hard-to-manage waste streams, seems to be especially promising for the implementation keratinases in leather and textile sectors of economy [18,19,75].

### 5.7. Environmental Protection

#### 5.7.1. Wastewater Treatment

Keratinases were studied in relation to treatment of heavily polluted, industrial wastewaters, such as molasses wastewater (MWW) from sugar factories, composed of various hazardous contaminants, including melanoidins [106,171].

Due to high concentrations of contaminants and co-pollutants, oxidation resistance and often significant antimicrobial effect, conventional secondary aerobic and anaerobic biological treatments are not efficient in decolorization of MWW [171]. Melanoidins, phenols and caramel are major colorants in treated MWW (TMWW), with the first group being the most abundant [171]. Some physical and physicochemical treatments (flocculation precipitation, membrane methods, photocatalysis), though yielding an excellent decolorization performance, are not economically and environmentally sustainable (high energy consumption, generation of secondary pollutants) [106]. However, cultivation of *Aspergillus citeromyces* and *Bacillus* strains on TMWW was applied in decolorization processes with oxidoreductases, such as laccase, glucose oxidase and lignin peroxidase, acting as main agents. Due to the need of co-enzymes and the requirements of specific conditions, oxidoreductases are often seen as unviable for large scale processes [106]. Therefore, hydrolases such as cellulases, cutinases and proteases have been widely studied for wastewater treatment, but removal of TMWW pigments has not been reported.

Several immobilized commercial hydrolytic enzymes, including lipase, α-amylase, protease, α-dextranase, pectinase, cellulase and keratinase (*Meiothermus* sp.) as well as immobilized oxidoreductases—laccase, glucose oxidase and manganese peroxidase—, have been tested for decolorization of TMWW from cane sugar factories. Additionally, recombinant keratinase from wild-type of *Meiothermus taiwanensis* WR-220 thermophilic bacterium (KMT-wt), expressed in *E. coli* has been immobilized on modified bagasse cellulose and also valorized. Commercial immobilized enzymes (500 U each) were added to 1000 mL of TMWW and left at 30 °C, pH 7.5 at 100 rpm for 12 h. After that, decolorization yields were calculated. Interestingly, commercial immobilized keratinase, eliminated from 86.6% to 91.1% of all colorants in used TMWW with other commercial hydrolases and oxidoreductases obtaining lesser yields. Similarly, immobilized KMT-wt (150 U) removed from 84.7% to 90.2% of all color contaminants after 2 h, and up to 85.2% of melanoidins present in TMWW. KMT-wt exhibited better chemical oxygen demand (COD) and biochemical oxygen demand (BOD) removal efficiency after continuous 5-day treatment than the commercial keratinase. However, it had little to no effect on caramel and phenols removal [106].

Taking into consideration high efficiency and lower costs of purchase of studied keratinases, studied enzymes could pose a potentially viable alternatives to available oxidoreductases [106].

#### 5.7.2. Prion Proteins Decontamination

Prions are infectious misfolded protein agents causing progressive neurological diseases known as transmissible spongiform encephalopathies (TSEs) [172]. Scrapie in sheep and goat, bovine spongiform encephalopathy (BSE) in cattle (also known as “mad cow disease”), chronic wasting disease (CWD) in deer (often called “zombie dear disease”) and Creutzfeldt-Jakob disease (CJD) and kuru disease in humans are all the examples of TSEs [157,172,173]. As TSEs are incurable and mostly fatal, management of prion transmission has become a crucial challenge for the public health. The danger of prion transmission through consumption of contaminated food and fodder impeded the animal waste usage in fodder formulations [18].

Due to high resistance to proteolysis performed by conventional proteases and traditional chemical and physicochemical sterilization methods, the protease-resistant prion protein (PrP^Sc^) needs a special approach. Effective ways of prion decontamination include incineration, high-pressure hydrolysis (132°C for 18 min) and usage of concentrated chemicals, such as sodium hydroxide, hydrogen peroxide, phenolics, guanidine thiocyanate and peracetic acid [172,173,174]. However, those methods are not regarded as environmentally sustainable and often not suitable for decontamination of medical devices and materials or substrates that could be recycled and further processed into value added products. Over the years, several studies reported, that keratinolytic enzymes may be potentially applied in prion protein degradation [18,22,172,173,174], due to the fact that similar to β-keratin, accumulating PrP^Sc^ are also largely composed of fibers with β-pleated sheets.

The *B. licheniformis* N22 keratinase and *Pseudomonas aeruginosa* NCIMB 8626 biosurfactant formulation was found to partially degrade ME7 scrapie prions after 1 h at 50 °C and, when temperature was raised up to 65 °C, studied PrP^Sc^ were undetected (by Western blot analysis) after only 10 min of the process. Reportedly, sole *B. licheniformis* N22 keratinase (EF) could not fully digest ME7 prions at 65 °C even after 1 h. In such conditions, sample with both enzyme and biosurfactant led to a complete digestion of infectious proteins [173].

Purified keratinase MSK103 secreted by *B. licheniformis* effectively degraded PrP^Sc^ in homogenates of brain tissues infected with scrapie or BSE prions after 2 h at 50 °C, and degraded prions to the levels below the limits of used method, when time of decontamination was extended to 20 h. MSK103 keratinase could also perform PrP^Sc^ biodegradation in dry samples [172]. Recombinant *E. coli* keratinase from *Pseudomonas aeruginosa* KS-1 synergistically with γ-glutamyl transpeptidase (GGT) and glutathione (GSH) complex (producing cysteinyl-glycine, a strong disulfide bonds reducing agents) demonstrated effective degradation of prion-like protein Sup 35NM in 15 min at 37 °C and pH 7.0 [174] Lesser effect was observed when *E. coli* keratinase was replaced by proteinase K. No to little effect was noted when enzymes were not accompanied by γ-glutamyl transpeptidase [174].

These enzymes showed the ability to degrade the PrSc protein into harmless and immunochemically undetectable forms. Therefore, keratinases, when mixed with other enzymes or biomolecules, can be used as potentially safe, more sustainable, and environmentally friendly method of decontamination and disposal of hazardous waste from infected animals, recycling nutrients in animal agriculture or cleaning medical equipment.

#### 5.7.3. Wild-Life Protection

As previously mentioned, keratinolytic enzymes can become a tool for stress assessment in industrially bred poultry, by determining levels of avian glucocorticoid stress hormone concentration deposited in feather tissue, as CORT hormone levels in epidermal keratin tissue corresponds with its concentrations in bloodstream during their period of growth [154,155].

Development of keratinase-involving methodology for improved steroid (glucocorticoids) extraction from chicken feathers [154] opened a way for implementing such approach towards samples of various epidermal tissue types of other species [175]. CORT tissue-deposited concentrations have been studied using *B. licheniformis* keratinase for sample digestion in whale (*Balaenoptera musculus*, *Balaena mysticetus* and *Eubalaena australis*) baleen, purple martin (*Progne subis*) feathers, arctic ground squirrel (*Urocitellus parryii*) hair, narrow-headed garter snake (*Thamnophis rufipunctatus*) and tegu lizard (*Salvator merianae*) shed skin and short-beaked echidna (*Tachyloglossus aculeatus*) spine, covering several vertebrate taxa [175]. A similar approach was also used to determine levels of reproductive hormones, progestagen and androgen in Temminick’s pangolin (*Smutsia temminickii*) scales [175,176]. As study shows, incorporation of keratin enzymatic digestion into standard protocol (after mechanical disruption and before organic solvent extraction) allows to obtain higher yields of CORT hormone from almost all samples. Relatively non-invasive, simple sample collection and possibility of analysis samples of limited mass and availability, seems to be especially beneficial in studying wild populations of endangered species [175,176]. However, differently than in industrially breed livestock, wild-life populations, notably animals leading mostly solitary lifestyle, may experience many stress-inducing periods unknown to researchers, which are impossible to detect or to catalogue during sample gathering and observational studies. Such obstacles can negatively affect comparability of hormone levels of certain individuals deposited in respective tissues [175,177]. The egg-laying process has been proven to rise CORT levels in blood and feather samples of red-legged partridge females (*Alectoris rufa*) with positive correlation between clutch sizes and hormone levels [155], posing theories that raised CORT concentration might be a natural cost of energy invested in reproduction or that the CORT hormone is needed for recovery after such a period [155].

Though promising, enzymatic digestion of keratin substrates possesses several concerns and limits [154,175,176]. As keratinases are proteolytic enzymes of diverse substrate specificity, not all enzymes are suitable for degrading a wide range of sampled tissues. Keratinase from *B. licheniformis* (FEED-0001; Creative Enzymes, Shirley, NY, USA) resulted in better yields of CORT hormones in hair, spine, baleen and feather samples varying from 30% to up to 377% higher hormone levels than in corresponding samples that were not subjected to enzymatic treatment. The highest change of CORT levels occurred in feather and hair samples, respectively. However, little to no change was recorded for snake and lizard shed skins samples. This may be due to many factors, e.g., sample age and composition, mainly α- and β-keratins ratios (very important in context of enzymatic digestion), distribution of hormones and their metabolites throughout sample, species differences, as well as sex and age of an tested individual [175,178]. Reportedly, CORT hormone and its metabolite concentrations can also differ based on body region that the sample was taken from [176]. As these differences seems to be species-specific, wildlife researchers should also take such premises into consideration when developing protocols for hormone assays deposited in keratin tissues [176,177].

## 6. Conclusions

Presented review explored various applications of keratinases and keratinolytic microorganisms in diverse industrial sectors, from environmental protection (decontamination of prion proteins, wastewater treatment, wildlife protection) to pharmaceuticals and medicine (anti-amyloid-β agents and transdermal drug delivery systems). Bioconversion of animal-derived keratin waste into biofertilizers, plant-growth stimulants and animal feedstuffs is in line with, at least partially, the enclosure of cross-sectoral production cycles, and caters to circular economy and bioeconomy models and policies. Application of keratinolytic microorganisms and enzymes, though still in a need of further investigation, gives an opportunity for the development of more sustainable methods of keratin waste management with generation of potentially high-end value-added products.

## Figures and Tables

**Table 1:** Keratin types characteristics [21,23,24].

Characteristic	α-Keratin	β-Keratin	γ-Keratin
Predominant affiliation	mammals, then birds, reptiles, fish and amphibians	mainly birds and reptiles with mammalian exceptions	mammals
Tissues	epidermis, fibers, nails, hooves, horns, mucus,	feathers, claws, beaks, scales, cuticle, epidermis	epidermis, fibrils cortex and fiber matrix
Form	filaments	filaments	globular
Main secondary structures	mainly α-helixes	mainly β-sheets	not determined
MW [kDa]	40–80	10–22	7–35

**Table 2:** Comparative analysis of various physicochemical treatment methods with their advantages and disadvantages [23,24,28,31,32,33,36].

Treatment Method	Physicochemical Factor	Advantages	Disadvantages
Solubilization	Organic solvents: DMF and DMSO	Powder form product, inhibition of microbial further product degradation, high efficiency process	Precipitation with acetone and drying is necessary, high organic solvent utilization
	Ionic liquids: [BMIM]Cl, [BMIM]Br		Precipitation is necessary (e.g., with acetone or methanol), high cost of ionic liquids, more difficult and less efficient extraction of keratin
Hydrothermal	Steam (80–140 °C; 10–15 psi)	No organic solvents consumption, possible acceleration by acid or base addition	Degradation of thermally unstable amino acids—Gln and Asn; the addition of a base results in additional degradation of Lys, Met, Tyr, Cys; Cys turn into to lysinoalanine and lanthionine; heating proteins leads to racemization of free and bound L-amino acids
Chemical reduction	Thioglycolate, DTT, β-ME, sodium sulfite and bisulfite in combination with high concentrations of urea, thiourea or surfactants	Efficient hydrogen bonds breaking, formation of poorly water-soluble kerateines with thiols and sulfonates at the site of disulfide bonds; no need for precipitation; cross-linking possibility after adding an oxidant, suitable for self-organizing biomaterials production	Significant consumption of chemical reagents, the process is usually carried out in an alkaline environment
Chemical oxidation	Peracetic acid or peroxycarboximidic acid	Formation of keratoses with sulfonic acid and cysteic acid groups instead of disulfide bonds; keratoses are hygroscopic, water-soluble and non-crosslinkable	Lower stability of keratoses compared with kerateins, no possibility of recreating disulphide bridges under oxidative conditions
Alkaline hydrolysis	Strong alkali, temperature 70–80 °C	Highly efficient degradation method	Degradation of Asn, Gln, Arg, Ser, Thr and Cys, formation of lysinoalanines and 8-aminoalanines, racemization of released and bound amino acids; use of concentrated bases
Acid hydrolysis	Strong acids—concentrated sulfuric acid, hydrochloric acid, high temperature	Breaking the hydrogen bonds increases the content of amorphous keratins	Degradation of Ser, Thr, Tyr, Cys, conversion of Asn, Gln, Met, Trp; the use of concentrated bases; lower efficiency than in other physicochemical methods

[BMIM]Cl—1-butyl-3-methylimidazolium chloride; [BMIM]Br—1-butyl-3-methylimidazolium bromide; DMF—dimethylformamide; DMSO—dimethyl sulfoxide; DTT—dithiothreitol; β-ME—2-mercaptoethanol.

**Table 3:** Biochemical properties and potential applications of recently isolated keratinolytic enzymes (examples from 2016 to 2021).

Microbial Source	Protease Type	Substrate	Optimal Temp. [°C]	Optimal pH	MW [kDa]	Potential Application	Essential Additives	Ref.
*Bacillus thuringiensis* MT1	Metallo	Keratin	50	9	80	Hair degradation	Ba^2+^, Ca^2+^, Mg^2+^, Mn^2+^	[25]
*Bacillus licheniformis* ALW1	n.a.	Azokeratin	65	8	n.a.	Feather degradation	n.a.	[50]
*Bacillus subtilis* PF1	n.a.	Casein	60	9	n.a.	Detergent formulation	Ca^2+^, Triton X-100, DMSO	[51]
*Bacillus* sp. CL18	Serine	Azocasein	55	8	n.a.	Recycling of keratin-rich waste	Ca^2+^, Mg^2+^, Triton X-100, DMSO	[52]
*Bacillus* sp. AD-W	Serine	Keratin azure	50	10	39	Recycling of keratin-rich waste	Mn^2+^, DTT	[53]
*Bacillus* sp. AD-AA3	Serine	Keratin azure	50	8	29	Recycling of keratin-rich waste	DTT, β-ME	[53]
*Bacillus subtilis* FTC02PR1	Serine	Azocasein	60	6−11	30	Feather degradation	SDS, Mn^2+^	[54]
*Bacillus haynesii* ALW2	n.a.	Keratin	70	8−9	n.a.	Hide dehairing	n.a.	[55]
*Bacillus amyloliquefaciens* S13	Serine	Keratin azure	50	6.5	28	Feather degradation, Hide dehairing	Ca^2+^, Mg^2+^, Zn^2+^	[56]
*Bacillus amyloliquefaciens* S13	Serine	Keratin azure	60	8	47	Feather degradation, Hide dehairing	Ca^2+^, Mg^2+^, Zn^2+^	[56]
*Bacillus subtilis* SCK6	Serine	Keratin	60	10	30.95	Skin dehairing	Cu^2+^, Co^2+^	[57]
*Brevibacillus parabrevis*	Serine	Keratin	60	8	28	Hide dehairing	Na^+^, Ca^2+^, Triton X-100, Tween-40	[58]
*Brevibacillus luteolum*	n.a.	Keratin azure	30	10	n.a.	Hair degradation	n.a.	[44]
*Caldicoprobacter algeriensis*	Serine	Keratin azure	50	7	33.25	Hide dehairing	Ba^2+^, Ca^2+^, Mg^2+^, Mn^2+^, Sn^2+^	[59]
*Meithermus taiwanensis* WR-220	n.a.	Chicken feather	65	10	30	Recycling of keratin-rich waste	n.a.	[60]
*Chryseobacterium aquifrigidense* FANN1	Metallo	Keratin azure	40−50	8	n.a.	Detergent formulation; feather degradation	Fe^3+^, Na^+^, Ca^2+^, Al^3+^, Triton X-100, Tween-80, SDS, DTT, DMSO, acetonitrile	[61]
*Streptomyces* sp. G11C	n.a.	Keratin azure	50	9	n.a.	Recycling of keratin-rich waste	n.a.	[62]
*Streptomyces* sp. CHA1	n.a.	Keratin azure	50	9	n.a.	Recycling of keratin-rich waste	n.a.	[62]
*Bacillus cereus*	n.a.	Azokeratin	n.a.	n.a.	n.a.	Transdermal delivery agent	n.a.	[63]
*Bacillus* sp. CSK2	n.a.	Keratin azure	60	8	n.a.	Bio-additive in detergents formulation	β-ME, DMSO, Tween-80	[64]
*Citrobacter diversus*	n.a.	Keratin azure	50	8.5−9.5	n.a.	Feather degradation	n.a.	[65]
*Arthrobacter* sp. NFH5	n.a.	Keratin	40	8	n.a.	Recycling of keratin-rich waste	n.a.	[66]
*Bacillus altitudinis* RBDV1	n.a.	Keratin azure	85	8	43	Recycling of feather waste	Mg^2+^, Mn^2+^, Ba^2+^, Zn^2+^, Fe^3+^, SDS, EDTA, DMSO, β-ME	[67]
*Bacillus cytotoxicus* LT-1	Metallo	Azocasein	40	7	n.a.	Scavenging activity feather protein hydrolysates	Zn^2+^, Ca^2+^, Mg^2+^, Mn^2+^	[68]
*Bacillus cytotoxicus* O11-15	Metallo	Azocasein	50	7	n.a.	Scavenging activity feather protein hydrolysates	Zn^2+^, Ca^2+^, Mg^2+^, Mn^2+^	[68]
*Bacillus cereus*	Serine	Casein	50	10	38	Degradation and recycling of keratin waste	Ca^2+^, Co^2+^, Mn^2+^, DTT	[69]
*Bacillus* sp. Nnolim-K1	Metallo	Keratin azure	60	8	n.a.	Feather degradation	β-ME, Tween-80, Ca^2+^	[70]
*Aspergillus stelliformis* AUMC 10920	n.a.	Keratin powder	50	8	n.a.	Degradation and recycling of keratin waste	n.a.	[71]
*Aspergillus sydowii* AUMC 10935	n.a.	Keratin powder	50	8	n.a.	Degradation and recycling of keratin waste	n.a.	[71]
*Fusarium brachygibbosum* AUMC 10937	n.a.	Keratin powder	50	8	n.a.	Degradation and recycling of keratin waste	n.a.	[71]

n.a.—not available; DMSO—dimethylsulfoxide; DTT—dithiothreitol; β-ME—β-mercaptoethanol; SDS—sodium dodecyl sulfate.

**Table 4:** Substrates used for proteolytic activity assay of keratinases.

Group	Substrate	Assay	References
Specific/dedicated substrates	Recombinant feather keratin	Absorbance at 280 nm	[79]
	Keratin azure	Absorbance at 595 nm	[74]
	Azo-keratin	Absorbance at 450 nm	[80]
	Azocasein	Absorbance at 366 nm	[81]
Natural substrates	Casein	Absorbance at 660 nm	[82]
	Cow horn	Absorbance at 280 nm	[76]
	Wool hair		[83]
	Feather		[84]
	Feather powder		[85]
	Human hair		[86,87]
Synthetic peptides	Suc-Ala-Ala-Pro-Phe-pNA	Absorbance at 405 nm	[88]
	Suc-Ala-Ala-Pro-Leu-pNA		[89]
	Bz-Arg-pNA		[88]
	Bz-Phe-Val-Arg-pNA		[90]
	Bz-Ile-Gly-Glu-Arg-pNA		[91]
	Suc-Leu-Leu-Val-Tyr-AMC	Fluorescent assay	[92]
	Leu-AMC		[93]
	Short peptides	Reverse-phase chromatography	[94]

**Table 5:** Chosen examples of engineering strategies for keratinolytic strain and/or enzyme improvement.

Keratinolytic Microorganism or Enzyme	Strategy	Result	References
*Bacillus* spp., *Meiothermus* sp., *Deinococcus radiodurans*	Heterologous expression of keratinase in *E. coli*, with pET vector	Enhanced production of desired enzyme; expression in less demanding, well-studied microorganism; possibility of expressing enzyme as His-tagged protein on C-terminal end (easy down-stream processing)	[60,119]
KerBP and KerBL in recombinant *E. coli* BL21 (DE3)	Switching of propeptide sequences between two different keratinases	Alteration of chaperoning activities; improvement of physicochemical KerBL characteristics	[120]
BsKER71	Heterologous expression of keratinase in *B. subtilis* WB600	Absence of homologous proteases; high keratinase production; less pathogenic tendencies than *E. coli*; higher enzyme activity	[121]
*Bacillus* spp.	Heterologous expression of keratinase in *P. pastoris*	Simple maintenance of host microorganism, possibility of cheaper inducers and secretion into the medium (α-factor), relatively simple downstream processing	[122]
Keratinase from *Streptomyces* sp. SCUT-3	Integrating native keratinase gene with an expression vector and transformation to native host	Controllable overexpression of an enzyme in native host; 5.6-fold increased keratinase production	[115]
Keratinase from *Bacillus amyloliquefaciens* K11		Controllable overexpression of an enzyme in native host; 6-fold increased keratinase production	[116]
*Brevibacillus* sp. ASS10II	UV-radiation random mutagenesis	Increased production of keratinase	[117]
*Candida parapsilosis*	Ethyl methanesulfonate random mutagenesis	Better keratin-degrading properties of engineered strain	[118]
*Bacillus subtilis*	Mutagenesis of sRNA genes, *codY* and *ccpA*	Improved expression of heterologous protein	[123]
*Bacillus licheniformis*	Antisense *aprA* deletion	Significant expression of protease *aprE* gene	[124]
*Bacillus subtilis ATCC 6051a*	Multigene deletion of intra- and extracellular proteases and RNases genes	Lower risk of heterologous genes and proteins degradation by native enzymes; increased expression of heterologous genes and increased production of desired enzymes	[125]

**Table 6:** Commercial biotechnological applications of keratinases [17,19,39,132].

Sector	Commercial Product	Manufacturer	Applications/Functions
**Cosmetic and skin care products**	Keratoclean Hydra PB	PROTEOS Biotech	Enzyme acts as an exfoliating, firming, thickening, moisturizing, anti-aging, anti-wrinkle, hair-removing and hair-growth-delaying agent
	Keratoclean PB	PROTEOS Biotech	Enzyme acts as anti-aging, anti-wrinkle, moisturizing, exfoliating, anti-hair growth and cell-renewing agent
	Keratoclean Sensitive PB	PROTEOS Biotech	Enzyme acts as anti-aging, anti-wrinkle, moisturizing, exfoliating and cell-renewing agent
**Agriculture**	Ronozyme^®^ ProAct	DSM/Novozymes	Enzyme improves digestibility and availability of proteins and amino acids (3–6% increase) in animal feed
	Cibenza DP 100	Novus International	Enzyme helps to increase availability of valuable nutrients for animal growth and performance, while minimizing negative effects of anti-nutritional factors and undigested protein in animal digestive systems
	Versazyme^®^	BioResource International, INc	Enzymes increase availability of energy, proteins, and minerals contained within fiber-rich cell walls or bound up in forms indigestible for livestock
	Valkerase^®^	BioResource International, INc	
**Medicine and medical treatments**	Prionzyme TM	Genencor International and Health Protection Agency	Engineered keratinase with increased activity, thermostability, and broader specificity for effective decontamination of medical instruments from prion proteins
	NATE-0853	Creative Enzymes^®^	Enzyme used for enzymatic treatment of cells, elementary body (EB) and glycosaminoglycans (GAGs) molecules, in the study of GAGs role of in the invasion of host cells by *Chlamydia pneumoniae* strains
	PURE 100 Keratinase	PROTEOS Biotech	Enzyme with wide range of supposed applications, incl. regulation of keratin concentration in pores for blisters and keratinized skin treatment, scars, dermatophytic and nail diseases treatment, as well as epithelial regeneration.

**Table 7:** Bioactive peptides—examples of groups, functions and sources [136,137,139].

Group	Representative/Sequence	Function	Source	
Angiotensin-I-Converting Enzymes Inhibitors (ACEI)	VPP	Lowering blood pressure, hypertension treatment	β-casein	Milk and dairy products
	IPP		κ-casein	
	YGLF; YLFF		β-lactoglobulin	
	ALPM (β-lactosin)		-	
	YAEERYPIL; IVF; ADHPFL; RADHP; FRADHPFL; RADHPF; LW		Ovalbumin	Eggs
	MNPKK (miopentapeptide A); ITTNP (miopentapeptide B)		Porcine myosin	Meat and meat derivatives
	MYPIGA		Porcine β-actin	
	GAXGLXGP; GAXGPAGPGGIXGERGLXG; GLXGSRGERGERGLXG; GIXGSRGERGPVGPSG		Chicken legs collagen	
	AAATP		Spanish-dry cured ham	
	VY		Sardine meat	Fish
	LKPNM		Smoked sardine meat	
	VW; GF; FG; SF; VY; YA; YG		Snail by-product	Gastropods
	VLIVP; YLAGNQ; FFL; IYLL; VMNKPG		Soy proteins	Plants
	IY; RIY; VWV; WIS		Rapeseed proteins	
Antioxidant peptides	VKEAMAPK; AVPYPQR; KVLPVEK; VLPVPEK	Free radical scavenging activity	β-casein	Milk and dairy products
	YFYPEL		α_s1_-casein	
	DAQEKLE; DSGVT; IEAEGE; EELDNALN; VPSIDDQEELM		Pork myofibrils	Meat and meat derivatives
	FPLEMMPF		Pollock meat proteins	Fish
	YAEERYPIL		Ovalbumin	Eggs
	LLPHH		Soy β-conglycinin	Plants
Antimicrobial peptides	Isracidin (1–23 fragment of α_s1_-casein)	Antibacterial, antifungal or antiviral activity	α_s1_-casein	Milk and dairy products
	165–203 fragment of α_s2_-casein		α_s2_-casein	
	184–209 fragment of β-casein		β-casein	
	Kappacine (106-169 fragment of κ-casein)		κ-casein	
	LDT1; LDT2S-S; LDCS-S		α-lactalbumin	
	Lactoferampin (268–284 fragment of lactoferrin)		lactoferrin	
	98–112 fragment of lysozyme		lysozyme	Eggs
	109–200 fragment of ovotransferrin		ovotransferrin	
	VTLASHLPSDFTPAVHASLD KFLANVSTVLTSKYR; TSKYR; STVLTSKYR; QADFQKVVAGVANALAHRYH		Bovine α-hemoglobin	Blood
	Nisin		*Lactococcus lactis*	
	Bacitracin		*Bacillus licheniformis*, *Bacillus subtilis*	
	Polymyxin B		*Bacillus polymyxa*	
	Tyrothricin (mixture of cyclic polypeptides)		*Bacillus brevis*	
	Gramicidin (mixture of gramicidin A, B and C)		*Bacillus brevis*	
Anti-amnesic peptides/Prolyl Endopeptidases (PEP) Inhibitors	Fragments of β-casein	Inhibition of prolyl endopeptidase (e.g., dipeptidyl peptidase IV; DPP IV) potentially responsible (when in increased concentrations) for memory loss and cognition disturbance; potential co-treatment of neurodegenerative diseases and type 2 diabetes	β-casein	Milk
	HLPPPV		maize γ-zein	Plants
	LLSPWNINA		By-product	Sake production
	GPGSPGGPL; GPVGXAGPPGK; GPM(O)GPXGVK; GPVGPSGPXGK; GPAGPXGVXGL		Deer collagen	Meat and meat derivatives
Opioid peptides	YPFPGPIPNSL (β-casomorphin-11)	Ligands of opioid receptors; pain relief, relaxing properties, regulative towards libido, body temperature and appetite; potentially inducing psychological disorders	Bovine β-casein	Milk
	YPFPGPI (β-casomorphin-7)			
	YPFPG (β-casomorphin-5)			
	YLGYLE (90-96 fragment of α_s1_-casein		α_s1_-casein	
	Hemorphins		Β-chain of hemoglobin	Blood
Taste-active peptides	Gurmarin	Sweet, taste-suppressing peptide	*Gymnema sylvestre*	Plants
	DF-OMe (aspartame)	Sweet taste; common sweetener	Synthetic pathways	
	RP; RA; AR; RG; RS; RV; VR; RM	Salty taste, potential replacement of kitchen salt for people with hypertension and/or diabetes;	Fish proteins	Fish
	AQTQSLVYPFPGPIPNSLPQNIPPLTQ; GPFPVIPPVAPPEVPGK; PALPEYLK; RGPPFIV; VYPFPPGINH; cyclic LWLW	Bitter taste	Bovine casein	Milk
	KGDEESLA	Umami taste	Bovine broth	Meat and meat derivatives
	GD; DE; EE; KG; GDG; AEA; VEV; DL; EEE		Various	
Micro- and macro-elements binding peptides	Caseinphosphopeptides (CPPs):	Metal-ion-binding peptides; increasing bioavailability of Ca^2+^, Zn^+^, Cu^2+^, Mn^2+^ and Fe^3+^	α_s1_-casein	Milk and dairy products
	59–79 and 64–84 fragments of α_s1_-casein			
	1–21 and 46–70 fragments of α_s2_-casein		α_s2_-casein	
	1–5 fragment of β-casein		β-casein	
	147–153 fragment of κ-casein		κ-casein	
	FLDDLTD; ILDK	Calcium-binding peptides	Whey α-lactalbumin	
	IPAVFK; VYVEELK		Whey β-lactoglobulin	
	LPTGPKS	Iron-biding peptide	Shrimp proteins	Crustaceans
	Fish-bone phosphopeptide (FBP)	Calcium-biding peptide	*Johnius belengerii* bone proteins	Fish
Bio-surfactant peptides	Surfactin (cyclic lipopeptide)	Decreasing water’s surface tension (also acting as an antibiotic)	*Bacillus subtilis*	

A = alanine, C = cysteine, D = aspartic acid, E = glutamic acid, F = phenylalanine, G = glycine, H = histidine, I = isoleucine, K = lysine, L = leucine, M = methionine, N = asparagine, P = proline, Q = glutamine, R = arginine, S = serine, T = threonine, V = valine, W = tryptophan, OMe = methyl ester.

**Table 8:** Some plant cultivation promoting properties of microbial-derived keratin hydrolysates [34,146,147,148,149].

Microorganism or Enzyme	Keratin Waste	Plant	Plant Cultivation Promoting Properties
*Bacillus subtilis*	−	−	P solubilization, IAA and ammonia production, antifungal activity (hydrogen cyanide synthesis)
*Trichoderma asperellum* T50, *T. atroviride*	Feathers or wool	*Solanum lycopersicum* (tomato)	Proton pump activation, seedling stimulation
*Bacillus thuringiensis, Bacillus sphaericus*	−	−	Insecticidal activity (towards Phlebotominae subfamily)
*Bacillus cereus*	Feather	*Oryza sativa* (rice seeds)	Enhanced growth, increased shoot and root lengths,
*Streptomyces* sp.	−	−	P solubilization, IAA, amonnia and siderophore production, antifungal activity,
*Bacillus aerius* NSMk2	Feathers	*Vigna radiata* (mung bane)	IAA production, faster speed germination, increased amount of DNA, RNA and total protein in root tips, overall enhancement of plant development
*Thermoactinomyctes* sp.	Feathers	*Cicer arietinum* (gram seeds)	Faster seed germination, plant height improvement
*Stenotophomonas maltophilia*	−	−	IAA and ammonia production, inhibition of *Botrytis cinerea*, *Colletotrichum gloeosporioides*, *Fusarium oxysporum* and *Pythium ultimum* growth
*Chyrsobacterium* sp. RBT	Feathers	*Musa* spp. (banana)	Higher antioxidant potential of fruits (increased phenolics and flavonoids content)
Promatex^®^ (*Bacillus* spp.)	Wool	*Zea mays* L. (maize seeds)	Modification of cellulose, protein and phenolics content in maize leaves, antifungal activities
Esperase^®^ (*Bacillus lentus*)			
Valkerase^®^ (*Bacillus licheniformis*)			

**Table 9:** Examples of keratinolytic microorganism utilized for bioconversion of keratin waste into animal feed [135,153].

Microorganism and/or Enzyme	Keratin-Rich Waste	Bioconversion Conditions	Results
*Chryseobacterium sediminis* RCM-SSR- 7	Poultry feathers	50 g/L feathers, 30 °C, pH 7.5, 84 h	Higher digestibility, amino acids enrichment
*Chryseobacterium* sp. kr6		50 g/L feathers, 30 °C, 120 h	Higher biological value, digestibility and amino acids content
*Bacillus* sp. MPTK6		30 g/L feathers, 30 °C, pH 10, 48 h	Higher digestibility
*Bacillus subtilis* AMR		10 g/L feathers, 26 °C, pH 8.0, 144 h	Mixed with cornmeal (26% of hydrolysate), higher nutritional value
*Vibrio* sp. kr2		60 g/L feathers, 30 °C, pH 6.0, 168 h	Partial replacement of soybean protein in feed, higher digestibility and biological value than feather meal, supplementation of diet with methionine
*Bacillus pumilus* A1	Wool waste	50 g/L wool, 45 °C, pH 10.0, 48 h	Higher digestibility than un-treated wool waste
*Bacillus pumilus* A1	Milled feathers	50 g/L milled feathers, 45 °C, pH 10.0, 48 h	Improved growth of Wistar rats by 2.5% and 5% of hydrolysate to standard diet

**Table 10:** Keratinolytic microorganisms or enzymes with potential application in detergent, leather or textile industry.

Keratinolytic Microorganism or Enzyme	Substrate/Additive	Process Parameters	Industry	Ref.
*Bacillus pumilus* GRK	Commercially available detergents (CAD)	60 °C, 1 h	Detergent	[128]
*Citrobacter diversus*		30 °C, 1 h		[65]
*Bacillus cereus* IIPK35		35 °C, 0.5 h, 6 mg/mL CAD		[169]
*Chryseobacterium aquifrigidense* FANN1		30 °C, pH 8.0, 1 h, 0.7% (*w*/*v*) of detergent		[61]
*Bacillus subtilis* PF1		50 °C, 1 h		[51]
*Brevibacillus* sp. AS-S10-II		100 °C, 1 h		[47]
*Bacillus aerius* NSMk2		4 °C, 30 min−2 h, 1% CAD		[114]
	Goat skin	37 °C, pH 8.0, 15 h	Leather	
*Brevibacterium luteolum* MTCC 5982	Goat skin	−		[44]
*Bacillus subtilis* S14	Bovine skin	24 °C, pH 9.0, 9 h		[91]
*Caldicoprobacter algeriensis* TH7C1	Goat, sheep and bovine skins	30 °C, pH 8.0, 12 h		[59]
*Bacillus amyloliquefaciens* S13	Sheep, goat, bovine skin	30 °C, 10 h		[56]
*Brevibacillus parabrevis* CGMCC 10798	Goat skin	37 °C, 7 h		[58]
*Laceyella sacchari* YNH	Goat skin	37 °C, pH 10.0, 1 h	Leather	[73]
	Blood-stained fabric		Detergent	
	Chocolate-stained fabric		Detergent	
*Bacillus cereus*	Cow skin	37 °C, 16 h	Leather	[78]
*Pseudomonas* sp.				
Recombinant keratinase from *B. licheniformis*	Wool fabrics	50 °C, pH 8.5, 2 h, 0.5 g/L non-ionic surfactant	Textile	[103]
*Bacillus* sp. G51	Merino wool	50 °C, pH 8.0, 2 h		[83]
*Bacillus subtilis* 168 E6-5	Wool fabric	55 °C, pH 7.0, 1 h		[82]
*Streptomyces* sp. 2M21	Wool fabric	37 °C, pH 8.0, 1 h, 0.5 g/L non-ionic surfactant		[170]

## Data Availability

No new data were created or analyzed in this study. Data sharing is not applicable to this article.
